# Address to Graduating College

**Published:** 1882-04

**Authors:** James Leslie


### ADDRESS

### To the Graduating Class of the Ohio College of Dental Surgery, March 1, 1882.

BY JAMES LESLIE, D.D.S.

This is the thirty-sixth annual commencement of the Ohio College of Dental Surgery, and is to you, young gentlemen graduates, a most important event. This college began thirty-seven years ago, but the exercises we call commencement simply means that you are now born legitimately into the great family of educated dentists, whose relative work among men is to assist in the care, repair and preservation of human teeth. The mission and recognition of our profession is new, compared with the practice of the healing art; and, yet, Reginald Stuart Poole, in one of his lectures before the Royal Institution, said "that the physicians of Egypt, during the Saite period, divided into specialists, as aurists, oculists, dentists, and others for diseases of different parts of the body; and that the teeth of the mummies show the excellence of the dental practice which had discovered the art of stopping with gold;" so that instead of your specialty being new, it is as old as the medical practice of Egypt. That it has become, and is now, recognized as eminently worthy of being studied and practiced as a specialty, has been acknowledged by the foremost medical practitioners in this country and Europe. Within the last ten years the College of Surgeons, in London, realizing the necessity of a special education for the practice of dentistry, established a curriculum of study, on the completion of which the student is entitled to a degree; and by appropriate laws being passed, no person can practice unless duly authorized. The growing importance of your profession has been recognized by the International Medical Congress, held lately in London, by its Committee of Arrangements, establishing a section known as the "Dental Section." To men who have been laboring in this and other lands for the improvement of the profession, recognition and measures of this kind by the savans of the medical profession in an International Congress can only be appreciated by the very few who have never ceased to labor in behalf of a good work. The apparent increase of the decay of the human teeth and diseases of the mouth, justifies the necessity of your special pursuit, almost more than that of aurist and oculist, because such diseases are more numerous than those of the eye and ear; and although the medical profession generally has been remiss in discovering, and tardy in acknowledging, the necessity of your specialty; yet it is to medical men that we are indebted for giving it their special attention. Hunter, and Bell, and Naysmyth, in their immortal labors, opened up and revealed the science; but the patient and skillful labor to make available their discoveries was needed; and that the first dental college ever founded was established in the United States, for the proper training and instruction of the dental practitioner, is one of the most remarkable facts in the history of dental science and professional education. And, stranger still, that Cincinnati, almost forty years ago, in what was then the backwoods of the West, so many thousands of miles from the then great seats of learning in this and other lands, should have been so distinguished as to have in her citizens a few men who saw and realized the necessity of a special college for dental instruction. Two young men (friends they were), corresponded on this subject. Dr. Chapin A. Harris in the east, and Dr. James Taylor in the west, the result being that a college was begun in Baltimore just one year before the one I now have the honor to represent. And, gentlemen, it must ever be to you a melancholy pleasure when the thought comes up, that you formed his last class. We all miss the presence of the great leader, the eminent teacher, the accomplished dentist and the christian gentleman. We remember how his face beamed with pleasure on such occasions as he handed to hundreds he had taught their diplomas, thus attaining the only object he desired—to send out among men educated dentists. Permit me to say, friends, that the locality where we are now assembled is a famous one in the history of our city. Just to your right was our first graveyard, our first church building, our first literary college where we are, and our jail close by. There were no stores west of Main on Fourth Street forty years ago; but it was the headquarters for dentists' offices. It was on this square that Drs. James Taylor, Allen and Rogers had their offices. In Dr. Taylor's office the Dental College was determined upon, and by the aid of Drs. Rogers, Allen and Cook a charter was obtained, and that group of men founded the Ohio College of Dental Surgery. Their names will be gratefully cherished by every man who believes that an educated professional man is forever to be preferred to him who simply assumes that he is able to practice dentistry because he has been in a dentist's office for a few months. One of the leading convictions of our day is that a man or woman, to labor successfully in any profession or calling, must understand its science, and the uses of its tools. The sculptor has a tender regard for the block of marble from which the bust and face of his friend or ideal image is to appear. It is a great matter to him whether it is hard or soft, pure white throughout or colored with streaks. When he gets the pure Cararra, and his tools in order, he carefully labors, chips and polishes till his work is done. To the sculptor that is dead matter. You may think so, but let me say you will never apply an instrument to a human tooth with more care and gentleness than he does when he is approaching that part of his work where the delicate risings leave or mark the gentle depressions which at once form the pliant muscles, bringing out the lifelike expression of the face. When engaged in your practice you will be called upon to operate on matter living and dead; for we say sometimes that a tooth is dead, but it is still surrounded with soft tissues and has a sensitiveness unsurpassed by any part of the body. To teach the people the value of a tooth, and the necessity of its preservation, is one of the most important duties you owe to the community where you may locate. Few people realize that in every one of the thirty-two teeth the Creator designed for the mastication of our food, each of them is accompanied with a delicate nerve; and the thirty-two exquisitely sensitive hair-like threads rise up distinct from the foramen of each tooth, then meet, forming larger branches, and all like so many telegraph wires saying nothing when at *ease*, but what a painful ticking is set up when diseased. All kinds of diseases were common in Burns' day, but an aching tooth he considered the "hell of them a'." I venture the opinion that if there had been dentists in his day as we have now he would not have so prominently connected this single fact of suffering with the bad place, and the witches that chased his beloved Tam and his mare Meg to the Brig o' Bonnie Doon. The dentist now can give immediate relief. In his day inflammation ran, as now, its length, mollified by fomentation, poulticing, assisted by the barber, and ending often by the blacksmith who had some pincers, which by main force the painful irritant could be extracted. I remember when an instrument was introduced for removing the molar teeth, which was simply the application of the lever power.

The same device is used every day, and can be seen in any lumber-yard where logs are being moved. It is simply a lever with a swivel hook attached. This instrument was adopted on a small scale, and, strangely, called a turnkey. It may be none of you have ever seen one, but I have a vivid recollection of a prominent dentist using that instrument many years ago; and it was considered a good thing, and a decided improvement over the blacksmith's tongs. Now there is not a single tooth but what has an instrument that is specially adapted for its extraction when necessary. It is just at this point in the history of the profession that a knowledge of the soft tissues and the connecting nerves demands the necessity of not only a knowledge of general anatomy but a perfect familiarity with the anatomy of the head. The muscles, nerves and blood vessels in their connection and relation to the teeth, became an absorbing theme with the few, and lifted the practice of dentistry at once to the plane of a medical specialty. To extract a tooth is a small matter, but to save it, rendering it still useful, is now the highest attainment of the profession.

The æsthetic students of our day that are reveling in sunflowers, lilies, and ceramics, might profitably spend some time in studying the nature and uses of our own ivories, as many people never make any inquiry about them, till they are compelled to come to the dentist for relief. I have met with persons who never gave any thought about their teeth, who were surprised when I told them that only one half of their face moves in the act of eating or speaking; and I have seen them put up their hand to see if that was true, and which half it is that moves. That is simply the knowledge of them in the mass, but the knowledge of how they appear, and grow and may be preserved is a most valuable part of that education which you can and should give whenever you have an opportunity. Indeed, I believe if you were to get up a lecture with suitable illustrations, it would be an admirable method of giving instruction, and prepare your patients to co-operate with you in practical operations. Our audience is about four hundred, and I presume none of us have the toothache; but I will venture to say that if every person here were to place themselves under the care of a competent dentist, he, with several assistants, might be kept busy for one year, so insidious is the decay of the teeth, and that too without giving any pain. True the occasional tick of the nerve is a gentle reminder that its bony covering is being invaded, but it has occurred so often, and passed away so quickly, the many timely warnings have been ignored, and a degree of pain is necessarily experienced, which might have been entirely avoided, had the gentle admonitions been attended to. The Psalmist declares, "Thine eyes did see my substance yet being imperfect, and in thy book all my members were written which in continuance were fashioned when as yet there was none of them." What a wonderful savan David must have been to have risked his reputation if he had not been inspired when he made known the wonderful fact that the members of our body were *fashioned in continuance* when as yet there was *none of them.* In view of this statement, how impressive is the fact that the formation and full development of the last tooth completes the physical man, and is the final product of that same law of continuance and marvelous creative skill of Him who said "Let us make man in our image." Every other part and organ of our body appears at birth, and the days as they pass mark the growth of the child in all its parts. Some years may pass away before we get our first set of teeth coming one or two at a time, and by the unerring law of *God's evolution from his involution*, we are provided with a set of twenty. But after some years that set is removed, and by virtue of this same law of continuance we are secured thirty-two teeth—the last one making its appearance when we are about twenty years of age, commonly known as the wisdom tooth. Every organ of the body God makes but once, but he provides two sets of teeth. I have not time to trace the history of their formation, and forbear to speak of cells, pabulum, protoplasm, lime, salts, and the marvelous process of crystalization in the formation of a tooth. You have heard a good deal about how they are fashioned in continuance, but the co-ordinating power that guides these moving cells so many years, and finally compels them to take their relative positions in regular order, so admirably adapted for the work designed, of speech and mastication, should impress us with a tender regard for their health, and that they are to each of us the last thing about us that marks the handiwork of God in the full development of the physical man. Magnify your office, then, among men, by giving them intelligent explanations of these pearls of the face, the perfection of which adds so much to its beauty, and the health of every other organ.

We are about to hand you a diploma that will secure for you the most favorable consideration by every practitioner; and travel where you may from here to China, you may find somewhere on the way graduates of your Alma Mater; and we confidently expect that its honorable reputation will be maintained by you, with that *esprit de corps* that marks and distinguishes the true professional man. Not only must you be honest and faithful operators, doing the best you can for your patients; but you must read and study the standard works of your profession. When you locate endeavor to make the acquaintance of some one who may have on hand a stock of dental literature. What has been done and accomplished by others may be a sort of Materia Medica in your practice; and let me say there are thousands of good hints in the records of dental practice. *The first dental magazine ever issued was one of the first assistants of your Alma Mater, and was issued and edited by the noble man from which the college sprang—Dr. James Taylor. It was the first evangel of the profession to the world; and during all these years, it has appeared every month, and to-day lives and is edited by Prof. Jonathan Taft, under the old name of the DENTAL REGISTER. But you should be subscribers for several monthly periodicals so you may keep posted about the improvements of the day. To prolong the use of the natural teeth is your relative work among men; but I can not conclude without reminding you that the chief object of your life is not merely to relieve pain and enable your friends to speak clearly, and chew their food comfortably. The time will come, notwithstanding all our skill, when the grinders will cease, and they may be few. There is a *thinking vita propria*—a life from without that gives vitality to every organ; and it is to-day the verdict, after years of most searching and brilliant experiments by Tyndal and others, that this life is

*See Editorial.

not of spontaneous generation, but comes from the outside. Dana and Virchow and hosts of eminent scientists confirm the same old conviction. Virchow, at the Berlin convention, in opposition to Hackel and all his school, affirmed that man is something more than mere "Carbon, Oxygen, Hydrogen & Co;" and Carlyle says that the "Gospel of Dirt" can't satisfy the nobler parts of our nature, and after all his deep study displayed in his admirable essays on men and things, accompanied with a good deal of grumbling, he declares the chief end of man "Is to glorify God and enjoy him forever." You may discover new facts, but no discovery can ever set aside the great historical facts of Moses as a divine law giver, with or without his mistakes, and Jesus the Nazarene, the Christ of God, whose resurrection from the dead is the great demonstration for the continuance of this life beyond. I point you to his life among men, and his instructions, as the most perfect and loftiest morality ever taught; and let us never forget that while he healed all diseases, the preparation of heroic and choice spirits and their communion in that life to come was and is the end of all he did and said.

